# Survival after cancer diagnosis in a cohort of HIV-positive individuals in Latin America

**DOI:** 10.1186/s13027-018-0188-3

**Published:** 2018-05-08

**Authors:** Valeria I. Fink, Cathy A. Jenkins, Jessica L. Castilho, Anna K. Person, Bryan E. Shepherd, Beatriz Grinsztejn, Juliana Netto, Brenda Crabtree-Ramirez, Claudia P. Cortés, Denis Padgett, Karu Jayathilake, Catherine McGowan, Pedro Cahn, on behalf of CCASAnet

**Affiliations:** 1 Fundación Huésped, Pasaje Gianantonio 3932, C1202ABB Buenos Aires, Argentina; 2 Vanderbilt University School of Medicine, 1161 21st Ave. S A2200 Medical Center North, Nashville, TN 37232 USA; 3 Instituto Nacional de Infectologia Evandro Chagas, Fundação Oswaldo Cruz, Av. Brasil, 4365 - Manguinhos, Rio de Janeiro, RJ 21040-900 Brasil; 4 Instituto Nacional de Ciencias Médicas y Nutrición Salvador Zubirán: Unidad del Paciente Ambulatorio (UPA), 5to piso Vasco de Quiroga # 15 Col. Sección XVI Delegación Tlalpan; C.P, 14000 Mexico City, Mexico; 5 Fundación Arriarán, Santa Elvira 629, Santiago, Chile; 6 Instituto Hondureño de Seguridad Social, Barrio la Granja, Tegucigalpa Honduras, Hospital Escuela Universitario: Av La Salud, Tegucigalpa, Honduras

**Keywords:** Cohort studies, HIV, Cancer, Survival, Latin America, AIDS defining cancer, Non AIDS defining cancer

## Abstract

**Background:**

This study aimed to evaluate trends and predictors of survival after cancer diagnosis in persons living with HIV in the Caribbean, Central, and South America network for HIV epidemiology cohort.

**Methods:**

Demographic, cancer, and HIV-related data from HIV-positive adults diagnosed with cancer ≤ 1 year before or any time after HIV diagnosis from January 1, 2000-June 30, 2015 were retrospectively collected. Cancer cases were classified as AIDS-defining cancers (ADC) and non-AIDS-defining cancers (NADC). The association of mortality with cancer- and HIV-related factors was assessed using Kaplan-Meier curves and Cox proportional hazards models stratified by clinic site and cancer type.

**Results:**

Among 15,869 patients, 783 had an eligible cancer diagnosis; 82% were male and median age at cancer diagnosis was 39 years (interquartile range [IQR]: 32–47). Patients were from Brazil (36.5%), Argentina (19.9%), Chile (19.7%), Mexico (19.3%), and Honduras (4.6%). A total of 564 ADC and 219 NADC were diagnosed. Patients with NADC had similar survival probabilities as those with ADC at one year (81% vs. 79%) but lower survival at five years (60% vs. 69%). In the adjusted analysis, risk of mortality increased with detectable viral load (adjusted hazard ratio [aHR] = 1.63, *p* = 0.02), age (aHR = 1.02 per year, *p* = 0.002) and time between HIV and cancer diagnoses (aHR = 1.03 per year, *p* = 0.01).

**Conclusion:**

ADC remain the most frequent cancers in the region. Overall mortality was related to detectable viral load and age. Longer-term survival was lower after diagnosis of NADC than for ADC, which may be due to factors unrelated to HIV.

## Background

Since the beginning of the epidemic, human immunodeficiency virus (HIV) and cancer have been intimately linked. People living with HIV have an increased cancer risk in comparison to the general population, not only for AIDS-defining cancers (ADC) but also for several non-AIDS-defining cancers (NADC) including Hodgkin lymphoma, anal cancer, lung cancer, liver cancer and certain skin cancers [1–3]. In high-income countries, although ADC were the most prevalent malignancies observed in the early years of the HIV epidemic, NADC increasingly account for cancer morbidity in the era of widespread availability of combined antiretroviral therapy (cART) [4–9]. As life expectancy increases for persons living with HIV, long-term exposure to known cancer risk factors such as oncogenic viruses (hepatitis B and C, Epstein Barr [EBV], and human papillomavirus [HPV]) and tobacco, and aging itself have contributed to an increase in the occurrence of NADC [1, 4–6]. Particularly in resource-rich countries with broad access to cART, causes of death have similarly shifted from AIDS-related conditions to other diseases [10, 11]. Cancer, particularly NADC, has become one of the most important causes of death in HIV-positive adults receiving cART [4, 7, 9, 12].

Several studies from high-income settings have examined predictors of mortality following cancer diagnosis in adults living with HIV. While cART use is associated with improved survival following ADC, it has not been associated with increased survival following NADC diagnosis [13, 14]. Additionally, CD4 count at cancer diagnosis has been less consistently associated with survival in patients with NADC [13, 15]. Poorer survival following NADC has also been associated with behavioral factors such as intravenous drug use and smoking in HIV cohorts [13, 15]. For some NADC, HIV- positive persons are more likely to be diagnosed at more advanced stage and may be less likely to receive standard chemotherapy compared to their uninfected peers, resulting in worse outcomes [13, 14, 16].

As cART has become increasingly available in Latin America, several studies have shown a growing prevalence of non-AIDS causes of morbidity and mortality, including cancer related deaths [15–20]. However, ADC remain frequent, and were the most common cause of cancer observed in a previous study from the region [20, 21]. Survival after cancer diagnosis has been described for resources-rich settings but there is a paucity of data from low- and middle-income countries, including Latin America, where cancer epidemiology and treatments may differ throughout the region [22–25]. This study aimed to describe cancer frequency and survival among HIV-positive individuals in Latin America. We also aimed to identify HIV clinical factors associated with survival following cancer diagnosis.

## Methods

### Cohort description and population

The Caribbean, Central and South America Network for HIV Epidemiology (CCASAnet) includes HIV clinical sites from seven countries (Argentina, Brazil, Chile, Haiti, Honduras, Mexico and Peru) and constitutes part of the International Epidemiologic Databases to Evaluate AIDS (IeDEA) [26]. Five sites contributed data to this study – Argentina (Fundación Huésped/Hospital Fernández [FH/HF], Buenos Aires, Argentina); Brazil (Instituto Nacional de Infectologia Evandro Chagas, Fundação Oswaldo Cruz, Rio de Janeiro, Brazil); Chile (Fundación Arriarán [FA], Santiago, Chile); Honduras (Instituto Hondureño de Seguridad Social [IHHS] and Hospital Escuela Universitario [HE], Tegucigalpa, Honduras); and Mexico (Instituto Nacional de Ciencias Médicas y Nutrición Salvador Zubirán [INNSZ], Mexico City, Mexico). HIV-positive individuals aged ≥18 years with at least one cancer diagnosis no more than one year before the date of HIV diagnosis or any time after HIV diagnosis and occurring between January 1, 2000 and June 30, 2015 were included in this study. HIV diagnosis date was considered as the reported date of HIV diagnosis, independently from the cancer diagnosis. Patients were excluded if they had an undetectable viral load at cART initiation, suggesting likely inaccurate cART data. Cancer cases were validated and categorized retrospectively as ADC (Kaposi sarcoma, non-Hodgkin lymphoma, invasive cervical cancer) or NADC (all other cancers) according to the CDC definition [27].

### Data management

Demographic, clinical, and laboratory data were collected at each site, de-identified, and sent to the CCASAnet Data Coordinating Center at Vanderbilt University (VDCC), Nashville, TN, USA, for data harmonization and processing. The data were checked for internal consistency and missing data, and quality assessments, including onsite audits, were performed. Institutional Ethics Review Boards from all sites and Vanderbilt approved the project, waiving the requirement for individual patient informed consent.

### Study outcomes

The primary outcome was time from cancer diagnosis to death due to any cause.

Death was ascertained by different means at the different sites. At IHSS/HE-Honduras, death was recorded when field workers were notified by family members after a call due to patients missing a visit. At all other sites, relatives of patients informed staff of the death (unless it occurred, and was already recorded, at the hospital), and in addition, study staff checked government death registry databases at least annually for subjects lost to follow-up for the FIOCRUZ-Brazil, FA-Chile, and INNSZ-Mexico sites. Patients were considered to be lost to follow- up if their vital status was unknown, and they had no clinical visit within the year prior to the database closing date at their site [28]. Closing dates for the databases were February 11, 2014 for FH/HF-Argentina, January 5, 2015 for FIOCRUZ-Brazil, August 11, 2014 for FA-Chile, October 29, 2015 for IHSS/HE-Honduras, and May 14, 2015 for INNSZ-Mexico.

### Statistical analysis

Demographic and clinical characteristics at the time of cancer diagnosis were summarized by site using median (Interquartile Range [IQR]) or percent (frequency), as appropriate.

The association of mortality with potential risk factors was assessed using Kaplan-Meier curves (overall, by cancer type, and by clinic site), log-rank tests, and Cox proportional hazard models, stratified (i.e., separate baseline hazards estimated) by site and type of cancer (ADC or NADC). Unadjusted models as well as multivariable models were fit. Additional analyses fit separate models for ADC and NADC. To investigate whether risk factors for mortality differed by type of cancer, separate analyses included type of cancer as a covariate and examined interaction terms between type of cancer and other predictor variables. Covariates included in the Cox models were selected a priori and included age, CD4 count (square root transformed), and plasma HIV-1 RNA level (VL) dichotomized to detectable versus undetectable using the threshold of 400 copies/mL at the time of cancer diagnosis, sex, timing of the cancer diagnosis relative to cART initiation (before/on vs. after cART initiation), and years from HIV diagnosis to cancer diagnosis. Baseline CD4 count was the closest non-missing value within a window 180 days before to 30 days after the date of cancer diagnosis. Baseline VL was considered the closest non-missing value using a window of 180 days before to 7 days after the date of cancer diagnosis. CD4 was included in models using restricted cubic splines to avoid assuming a linear relationship with the outcome. Missing data were present for CD4 (31%), VL (46%), and years from HIV diagnosis to cancer diagnosis (1.4%). Multiple imputation, using five imputation replications, was used to account for missing data in the multivariable models. All analyses were performed using R statistical software, Version 3.3.0 (https://www.R-project.org). Analysis code is posted at http://biostat.mc.vanderbilt.edu/ArchivedAnalyses.

## Results

Among 15,869 eligible adult patients (FH/HF- Argentina: 4912, FIOCRUZ-Brazil: 5807, FA-Chile: 2476, IHSS/HE-Honduras: 1326, INNSZ-Mexico: 1348), 954 (6%) had at least one cancer diagnosis, of which 783 (5%) were eligible for this analysis. Of those patients excluded, 126 were diagnosed with cancer before the year 2000, 23 were diagnosed with cancer on the last day of their follow up, five had inaccurate cART information, 16 had an unknown date of cancer diagnosis, and one had cancer diagnosis at < 18 years old.

Table 1 shows the characteristics of patients with cancer diagnoses overall and by site. In all sites except IHSS/HE-Honduras the majority of the patients were male. Median age at cancer diagnosis was 39 years (IQR 32–47); patients with cancer were older in FIOCRUZ-Brazil and IHSS/HE-Honduras (41 years) and younger in INNSZ-Mexico (34 years). Median time between HIV diagnosis and cancer diagnosis was 1.7 years (IQR 0.2–6.7), ranging from a median of 0.4 years in INNSZ-Mexico to 4.0 years in FIOCRUZ-Brazil. Median time between HIV diagnosis and cART start was 0.5 years (IQR 0.1–3.1), being longer for FIOCRUZ-Brazil (0.6) and FA-Chile (1.0) and shorter for IHSS/HE-Honduras (0.3) and INNSZ-Mexico (0.2). Median CD4 count at cancer diagnosis was 148 cells/μL (IQR 44–364), ranging from 82 cells/μL for patients in FH/HF-Argentina to 190 cells/μL in FIOCRUZ-Brazil. Forty-two percent of the patients were diagnosed with cancer before or concomitant to cART initiation, ranging from 36% in FIOCRUZ-Brazil and IHSS/HE-Honduras to 54% in INNSZ-Mexico. Approximately 4% of patients never started cART. Among the 306 patients on cART at cancer diagnosis with available viral load, 185 (60%) had an undetectable VL (< 400 copies/ml).

**Table 1:** Patients´ characteristics and cancer type by site

	Argentina	Brazil	Chile	Honduras	Mexico	Combined
	*N* = 156	*N* = 286	*N* = 154	*N* = 36	*N* = 151	*N* = 783
Sex (*n* = 783), % (n)						
Female	12% (19)	23% (67)	9% (14)	53% (19)	14% (21)	18% (140)
Male	88% (137)	77% (219)	91% (140)	47% (17)	86% (130)	82% (643)
Age at cancer diagnosis (*n* = 783), median (IQR)	38 (33–45)	41 (35–48)	39 (33–49)	41 (30–48)	34 (29–42)	39 (32–47)
Years from HIV diagnosis to cancer diagnosis (*n* = 772), median (IQR)	1.79 (0.10–5.94)	3.97 (0.25–9.95)	1.88 (0.38–7.05)	1.60 (0.41–4.69)	0.45 (0.06–2.77)	1.67 (0.18–6.74)
Years from cART initiation to cancer diagnosis (*n* = 749), median (IQR)	0.040 (− 0.080–2.49)	0.53 (− 0.02–5.86)	0.33 (− 0.04–1.93)	0.53 (− 0.04–2.30)	−0.010 (− 0.09–0.93)	0.19 (− 0.06–2.87)
Cancer diagnosis relative to cART initiation (*n* = 783), % (n)						
Cancer diagnosis after cART initiation	53% (83)	64% (183)	63% (97)	64% (23)	46% (69)	58% (455)
Cancer diagnosis before/at cART initiation/Did not start cART	47% (73)	36% (103)	37% (57)	36% (13)	54% (82)	42% (328)
CD4 count (cells/μL) at cancer diagnosis (*n* = 537), median (IQR)	82 (14–276)	190 (54–425)	171 (56–388)	176 (90–382)	141 (45–300)	148 (44–364)
HIV-1 RNA at cancer diagnosis (log_10_) (*n* = 422), median (IQR)	4.4 (2.6–5.3)	3.2 (2.6–4.9)	2.6 (2.6–4.9)	2.6 (2.6–4.4)	4.6 (2.6–5.3)	3.9 (2.6 5.0)
HIV1-RNA status at cancer diagnosis (cut point = 400 copies/mL), % (n)						
Undetectable	31% (16)	49% (80)	49% (41)	67% (6)	38% (43)	44% (186)
Detectable	69% (36)	51% (84)	51% (43)	33% (3)	62% (70)	56% (236)
Status at the end of follow-up (*n* = 783), % (n)						
Alive	81% (126)	61% (174)	67% (103)	61% (22)	84% (127)	70% (552)
Dead	19% (30)	39% (112)	33% (51)	39% (14)	16% (24)	30% (231)
Follow-up (years) (*n* = 783), median (IQR)	2.20 (0.55–6.13)	2.30 (0.66–4.91)	3.69 (0.62–8.87)	1.06 (0.59–5.42)	3.33 (0.92–5.84)	2.51 (0.69–6.13)
Type of cancer (n = 783), % (n)						
AIDS-defining cancers	82% (128)	66% (188)	70% (108)	72% (26)	75% (114)	72% (564)
Kaposi sarcoma	59% (92)	51% (147)	42% (65)	28% (10)	42% (64)	48% (378)
Non-Hodgkin lymphoma	20% (31)	13% (38)	27% (41)	19% (7)	22% (33)	19% (150)
Invasive cervical cancer	3% (5)	1% (3)	1% (2)	25% (9)	11% (17)	5% (36)
Non-AIDS-defining cancers	18% (28)	34% (98)	30% (46)	28% (10)	25% (37)	28% (219)
Anal	2% (3)	5% (15)	3% (4)	3% (1)	13% (19)	5% (42)
Breast	1% (2)	5% (15)	1% (1)	6% (2)	0% (0)	3% (20)
Colon	1% (2)	1% (3)	2% (3)	3% (1)	0% (0)	1% (9)
Hodgkin lymphoma	2% (3)	1% (4)	6% (9)	0% (0)	5% (7)	3% (23)
Lung	3% (4)	3% (8)	0% (0)	0% (0)	0% (0)	2% (12)
Prostate	0% (0)	2% (5)	1% (1)	3% (1)	3% (5)	2% (12)
Renal	1% (2)	2% (6)	0% (0)	0% (0)	0% (0)	1% (8)
Skin	4% (7)	7% (20)	5% (7)	6% (2)	1% (1)	5% (37)
Testicular	1% (1)	0% (0)	5% (7)	0% (0)	0% (0)	1% (8)
Other^a^	3% (4)	8% (22)	9% (14)	8% (3)	3% (5)	6% (48)

IQR interquartile range

^a^Other: acute leukemia (2–2%), bladder cancer (2–2%), brain cancer (5–6%), cancer with unknown primary (3–3%), chronic leukemia (1–1%), esophageal cancer (3–3%), eye cancer (1–1%), gall bladder (1–1%), gastric cancer (7–8%), laryngeal cancer (3–3%), not otherwise specified leukemia (3–3%), liver cancer (1–1%), multiple myeloma (2–2%), oral cancer (4–4%), ovarian cancer (2–2%), pancreatic cancer (1–1%), penile cancer (1–1%), sinus cancer (1–1%), soft tissue sarcoma (1–1%), thyroid cancer (4–4%)

As shown in Table 1, 564 of the 783 (72%) cancer cases were ADC, ranging from 66% in FIOCRUZ-Brazil to 82% in FH/HF-Argentina. Overall, Kaposi sarcoma (KS) was the most frequent cancer (48%), followed by non-Hodgkin lymphoma (19%) and cervical cancer (5%). Among NADC, anal cancer (42 cases, 5%) was the most common cancer followed by skin cancer (37 cases, 5%) and Hodgkin lymphoma (23 cases, 3%).

Table 2 compares characteristics of persons diagnosed with ADC and NADC. ADC were more likely to be diagnosed in males than NADC (86% vs 72%). Patients diagnosed with ADC were younger at cancer diagnosis than those diagnosed with NADC (median 37 vs 45 years), had a more recent HIV diagnosis (median 0.8 vs 5.3 years), had spent less time on cART (median 0.0 vs 3.1 years), had lower CD4 count at cancer diagnosis (median 89 vs 376 cells/μL), and were more likely to have a detectable VL (69% vs 30%). A higher percentage of NADC were diagnosed in the later periods of our study (65% of all NADC were diagnosed after 2008 vs. 52% of all ADC). Specifically, 12% (17 of 137), 29% (60 of 210), 32% (83 of 256), and 33% (59 of 180) of the cancers diagnosed during 2000–2003, 2004–2007, 2008–2011 and 2012–2015, respectively, were NADC.

**Table 2:** Patients' characteristics according to cancer type

	Non-AIDS-defining cancers (*n* = 219)	AIDS-defining cancers (*n* = 564)	Combined (*n* = 783)	P
Sex (*n* = 783), % (n)				< 0.001
Female	28% (61)	14% (79)	18% (140)	
Male	72% (158)	86% (485)	82% (643)	
Age at cancer diagnosis (*n* = 783), median (IQR)	45 (38–54)	37 (31–44)	39 (32–47)	< 0.001
Year of cancer diagnosis (*n* = 783), median (IQR)	2009 (2006–2012)	2008 (2004–2011)	2008 (2005–2011)	< 0.001
Years from HIV diagnosis to cancer diagnosis (*n* = 772), median (IQR)	5.26 (2.09–11.00)	0.82 (0.11–5.05)	1.67 (0.18–6.74)	< 0.001
Years from cART initiation to cancer diagnosis (*n* = 749), median (IQR)	3.08 (0.50–8.48)	0.02 (−0.08–0.86)	0.19 (−0.06–2.87)	< 0.001
Cancer diagnosis relative to cART initiation (*n* = 783), % (n)				< 0.001
Cancer diagnosis after cART initiation	79% (172)	50% (283)	58% (455)	
Cancer diagnosis before/at cART initiation/Did not start cART	21% (47)	50% (281)	42% (328)	
CD4 count (cells/μL) at cancer diagnosis (*n* = 537), median (IQR)	376 (229–573)	89 (29–230)	148 (44–364)	< 0.001
HIV-1 RNA at cancer diagnosis (log_10_) (*n* = 422), median (IQR)	2.6 (2.6–3.7)	4.6 (2.6–5.2)	3.9 (2.6–5.0)	< 0.001
HIV-1 RNA status (cut point = 400 copies/mL), % (n)				< 0.001
Undetectable	70% (99)	31% (87)	44% (186)	
Detectable	30% (42)	69% (194)	56% (236)	
Status (*n* = 783), % (n)				0.20
Alive	67% (147)	72% (405)	70% (552)	
Dead	33% (72)	28% (159)	30% (231)	
Follow-up (yrs) (*n* = 783), median (IQR)	2.17 (0.66–5.13)	2.62 (0.69–6.34)	2.51 (0.69–6.13)	0.18

Numbers after percentages are frequencies. IQR: Interquartile range. Tests used: Pearson test for categorical variables; Wilcoxon Rank Sum test for continuous variables

**Fig 1.**
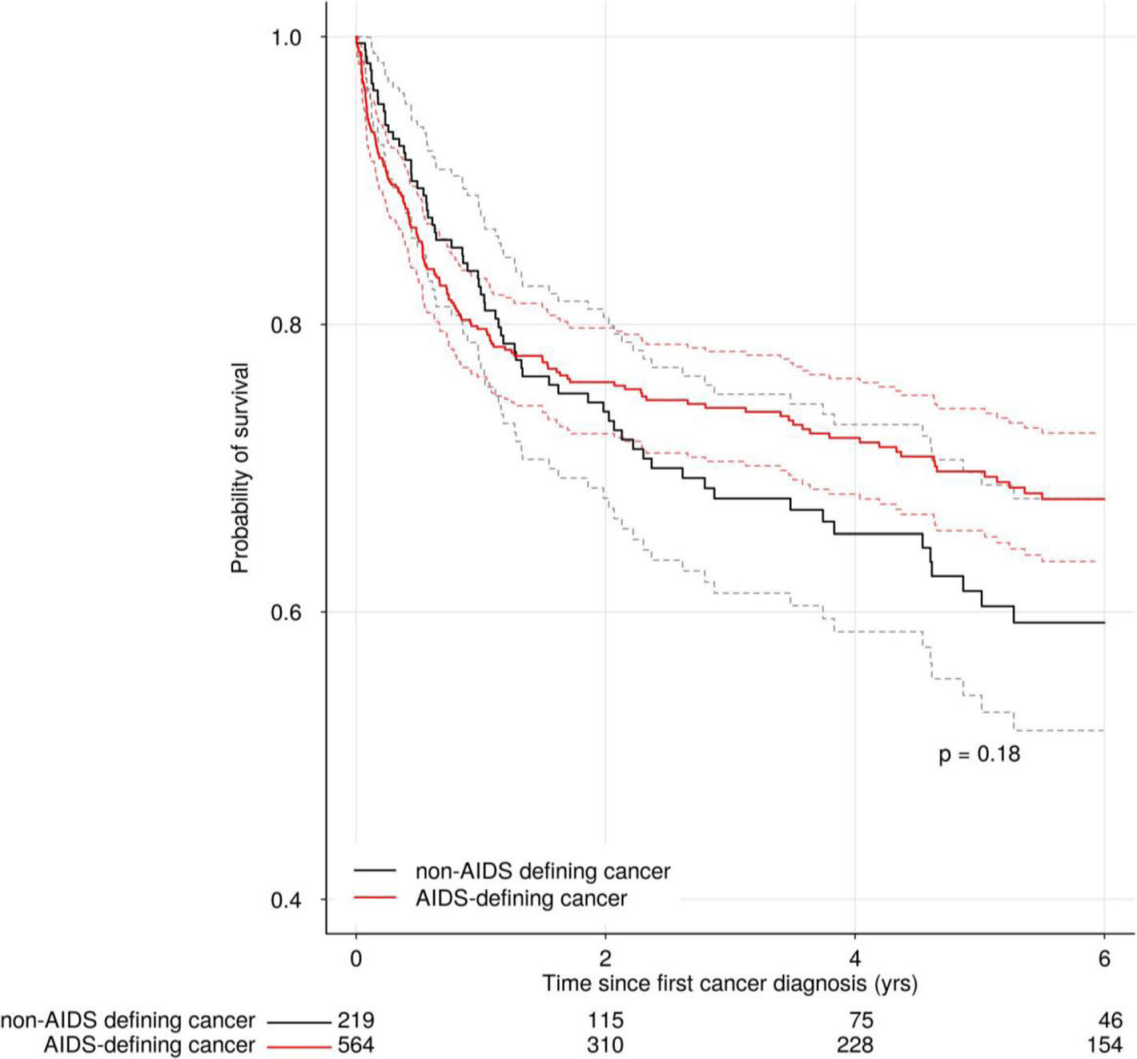
Survival curves for AIDS-defining cancers and non-AIDS-defining cancers

A total of 231 (30%) patients diagnosed with cancer died of any cause during the follow-up period; the median follow-up after cancer diagnosis was 2.5 years (IQR 0.7–6.1). Overall survival probabilities at 1, 3, and 5 years after diagnosis were 80%, 72%, and 67%, respectively. Survival probabilities for those with ADC and NADC are shown in Fig. 1.

**Fig 2.**
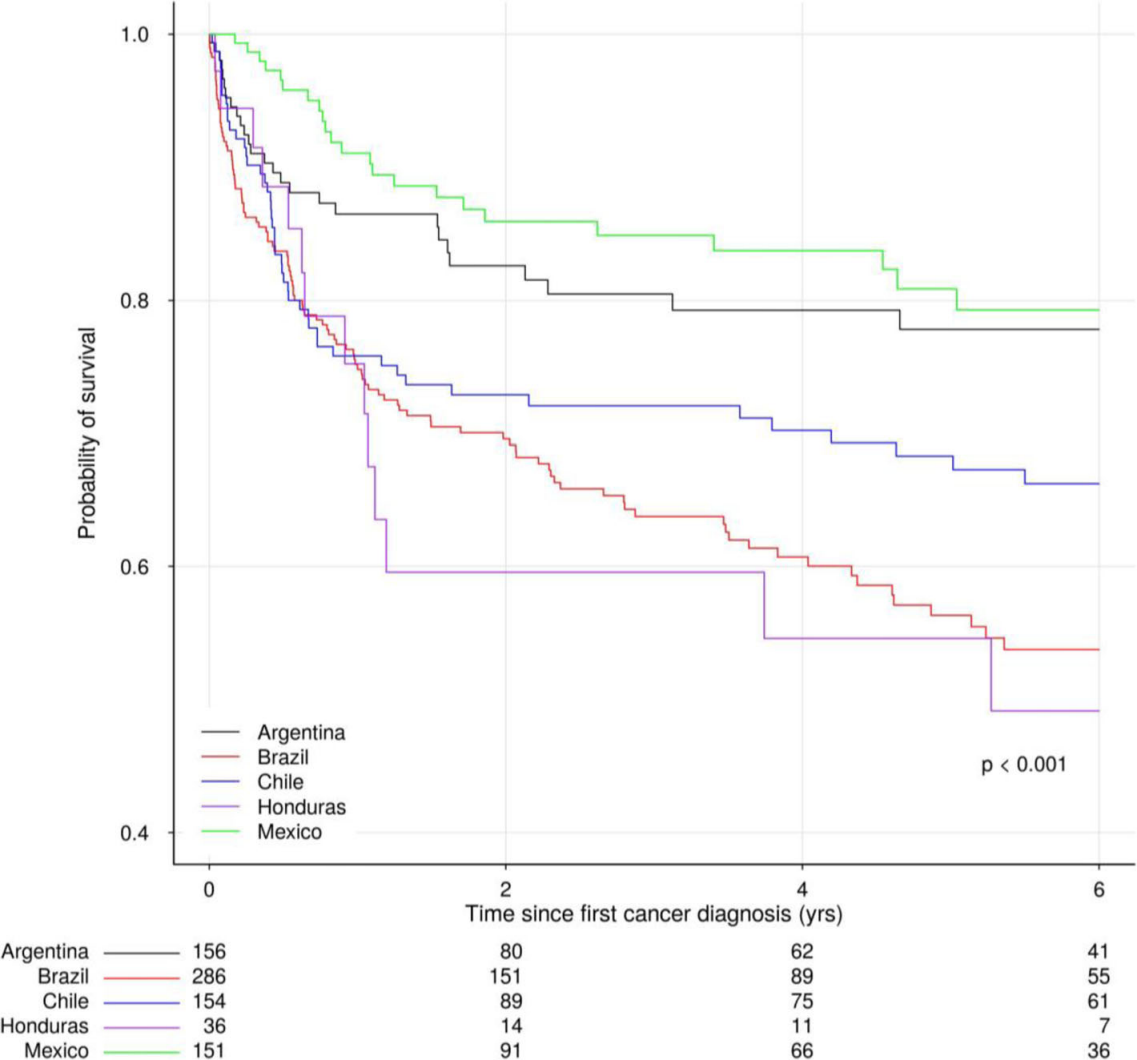
Survival curves after cancer diagnosis by site

Survival was initially higher for those with NADC than ADC, but at the end of one year it was similar (81% vs 79%), and at three and five years it was lower (67% vs 74% and 60% vs 69%, respectively). Overall survival curves did not statistically differ (*p* = 0.18). Survival after cancer diagnosis differed by site (*p* <  0.001); at five years it was estimated as 79% for INNSZ-Mexico, 78% for FH/HF-Argentina, 67% for FA-Chile, 56% for FIOCRUZ-Brazil and 49% for IHSS/HE-Honduras (Fig. 2).

Table 3 shows results from univariate and multivariable Cox proportional hazard models assessing associations between patient characteristics at cancer diagnosis and mortality, stratified by cancer type and clinic site. In the unadjusted analyses, patients with lower CD4 count tended to have higher hazards of mortality (18% higher for patients with 100 CD4 cells/μl vs 350 cells/μl), but this association became less pronounced after adjusting for other variables (*p* = 0.80). In the adjusted analysis, patients with a detectable VL at cancer diagnosis (≥ 400 copies/mL) had a 63% higher hazard of death than patients with an undetectable VL (95% confidence interval [CI] for adjusted hazard ratio [aHR] =1.08–2.47; *p* = 0.02). Similarly, age at cancer diagnosis and time from HIV diagnosis to cancer diagnosis were also significantly associated with mortality. Risk of mortality increased with age at cancer diagnosis (2% for each year; 95% CI 1.01–1.03; *p* = 0.002). From the time of HIV diagnosis, cancers diagnosed one year later were associated with a 3% higher hazard of mortality (95% CI 1.01–1.06; *p* = 0.01).

**Table 3:** Unadjusted and adjusted results from the Cox proportional hazard models investigating the association between time from cancer diagnosis to death, dichotomizing HIV-1 viral load

	Unadjusted			Adjusted		
Covariate	HR	95% CI	P	HR	95% CI	P
Sex			0.41			0.22
Female (ref)	1.00			1.00		
Male	0.87	(0.61–1.22)		0.81	(0.57–1.14)	
Cancer diagnosis relative to cART initiation			0.61			0.41
Cancer diagnosis after cART initiation (ref)	1.00			1.00		
Cancer diagnosis before/at cART initiation/Did not start cART	0.93	(0.70–1.23)		0.87	(0.62–1.22)	
CD4 count (cells/μL) at cancer diagnosis			0.06			0.80
100	1.18	(0.96–1.44)		0.99	(0.82–1.21)	
200	1.03	(0.91–1.17)		0.98	(0.86–1.12)	
350 (ref)	1.00			1.00		
500	1.03	(0.89–1.19)		1.03	(0.90–1.17)	
HIV-1 RNA at cancer diagnosis			0.21			0.02
Undetectable (ref)	1.00			1.00		
Detectable (> 400 copies/mL)	1.28	(0.87–1.88)		1.63	(1.08–2.47)	
Years from HIV diagnosis to cancer diagnosis	1.03	(1.01–1.06)	0.01	1.03	(1.01–1.06)	0.01
Age at cancer diagnosis (per year)	1.02	(1.01–1.03)	0.003	1.02	(1.01–1.03)	0.002

Results were fairly similar when ADC and NADC were considered separately (Table 4). Detectable viral load at diagnosis of an ADC was associated with an 83% increase in the adjusted hazard of mortality (95% CI 1.03–3.24; *p* = 0.04). In contrast, a detectable viral load at the time of NADC diagnosis was not associated with a higher hazard of mortality (aHR = 0.98; 95% CI 0.41–2.30; *p* = 0.96), although these hazard ratios did not statistically differ between ADC and NADC (*p* = 0.81, test for interaction). In general, there was little evidence that hazard ratios differed between ADC and NADC for any of the patient characteristics considered (*p* > 0.15 for all variables).

**Table 4:** Adjusted analyses according to type of cancer (AIDS-defining cancer and non-AIDS-defining cancer)

	ADC			NADC			p (test for interaction)
Covariate	HR	95% CI	P	HR	95% CI	P	
Sex			0.08			0.86	0.18
Female (ref)	1.00			1.00			
Male	0.67	(0.43–1.05)		1.05	(0.61–1.80)		
Cancer diagnosis relative to cART initiation			0.59			0.94	0.81
Cancer diagnosis after cART (ref)	1.00			1.00			
Cancer diagnosis before/at cART initiation/Did not start cART	0.90	(0.60–1.33)		1.03	(0.42–2.52)		
CD4 count (cells/μL) at cancer diagnosis			0.87			0.66	0.23
100	1.08	(0.75–1.55)		1.09	(0.81–1.46)		
200	1.04	(0.83–1.32)		1.01	(0.89–1.15)		
350 (ref)	1.00			1.00			
500	0.96	(0.79–1.18)		1.05	(0.90–1.22)		
HIV-1 RNA at cancer diagnosis			0.04			0.96	0.81
Undetectable (ref)	1.00			1.00			
Detectable (400 copies/mL)	1.83	(1.03–3.24)		0.98	(0.41–2.30)		
Years from HIV diagnosis to cancer diagnosis	1.04	(1.00–1.07)	0.03	1.02	(0.98–1.07)	0.26	0.71
Age at cancer diagnosis (per year)	1.02	(1.01–1.04)	0.005	1.02	(1.00–1.04)	0.12	0.70

The last column provides *p*-values comparing hazard ratios for ADC vs. NADC using tests for interaction

AIDS-defining cancer (ADC); non-AIDS-defining cancer (NADC)

*= *p*-value from the model on the full cohort with the type of cancer interacted with the given variable. There were 219 subjects in the NADC cohort and 72 deaths. Similarly, there were 564 subjects in the ADC cohort and 159 deaths

## Discussion

In this multisite cohort study of HIV patients diagnosed with cancer across Latin America, we found that age, time since HIV diagnosis, and detectable viral load were predictive of mortality after accounting for cancer type, sex, cART use, and CD4 count. While ADC were the most prevalent cancers diagnosed, an increasing proportion of NADC were diagnosed in more recent years. Despite marked clinical differences in patient characteristics at cancer diagnosis, there was no meaningful difference in survival in the first year after cancer diagnosis for patients diagnosed with ADC versus those diagnosed with NADC, though there was a suggestion that NADC may be associated with increased mortality 3 and 5 years after diagnosis. In this region of low- and middle-income countries, these results reflect the dynamics of cancer epidemiology in HIV positive patients with increasingly available ART and longer life expectancy.

Site was also significantly associated with mortality. This might reflect varying prevalence of the different cancers and differences in access to treatment and care.

We observed an increased hazard of mortality following cancer diagnosis associated with detectable viral load (63% higher than patients with VL < 400 copies/ml); this hazard was particularly higher (83%) for those diagnosed with an ADC. HIV VL has been demonstrated in previous studies to predict mortality in patients diagnosed with ADC, particularly HIV-associated lymphomas [29–31]. Virologic suppression has also been associated with improved survival in studies of NADC [31, 32]. In our population, lack of virologic suppression was associated with mortality after cancer diagnosis and was independent of any observed immunologic association with mortality, suggesting that viral control may be a marker of other patient or clinical characteristics associated with improved cancer outcomes rather than the immunologic effect of HIV. In KS, the most frequent ADC in our study, HIV-1 has a direct role in disease pathogenesis, due to pro-oncogenic effects of HIV-1-encoded proteins such as the Tat protein. Tat, a regulatory protein released by HIV-infected cells, protects cells from apoptosis, promotes the growth of spindle cells in synergy with inflammatory cytokines, [33–35] and contributes to the intense neoangiogenesis found in KS lesions [36].

Our study did not find that CD4 at the time of cancer diagnosis was predictive of mortality following cancer diagnosis in any of the analyses performed, which differs from studies in high-income settings [32, 37]. Though median CD4 count at cancer diagnosis was significantly lower among patients diagnosed with an ADC versus those diagnosed with a NADC, CD4 count among all patients was very low in our cohort (median 148 cells/μl, IQR: 44–364) and 42% of all the patients were not on cART at cancer diagnosis, suggesting late HIV diagnosis or access to care. The very low CD4 counts among most patients and the fact that the majority had ADC may have limited our ability to detect a meaningful role of immunosuppression and mortality risk. Another possible explanation may be related to the high prevalence of KS, which if diagnosed in early stages and treated, generally has a good prognosis.

Although our study was focused on survival, it also describes cancer epidemiology of HIV-positive adults in the Latin America region. Five percent of patients overall were diagnosed with cancer during the study period; the majority of cases were ADC, and KS was the most frequent. KS incidence is geographically variable, and depends on the prevalence of human herpesvirus-8 infection, the prevalence of HIV, and access to HIV treatment [38, 39]. Our findings are consistent with other studies where ADC were still the most frequent malignancies observed, even in more recent years of the epidemic [20, 21, 40–42]. However, many recent reports describe an increasing proportion of cancers due to NADC, and occurring more frequently than ADC, among HIV cohorts [24, 43–48]. The most frequent NADC observed in our cohort were anal cancer, Hodgkin disease, and skin cancer, which likely are related to coinfection with oncogenic viruses such as HPV and EBV, and an aging population [24, 49–51]. Lung cancer was less frequently observed in our cohort compared to other reports, [24, 52] possibly due to differing patterns of tobacco use, diagnostic capabilities, or case ascertainment, which needs further exploration. Anal cancer was more frequent in FIOCRUZ- Brazil and INNSZ-Mexico; possibly related to more frequent anal cancer screening practices. These trends will probably continue to change as HIV-positive individuals live longer and antiretroviral therapy is started earlier. Of note, a high number of testis cancer was found in FA-Chile. Data from the general population show that Chile has a higher incidence of testis cancer (age- standardized rate 6.8 per 100,000 population) than the other countries participating in the study (ranging from 0.4 in Honduras to 5 per 100,000 population in Argentina) [53]. Further investigation will be needed to determine whether there are particular associated factors in the HIV-positive population.

Across our region, cancer trends and mortality differed, likely reflecting differences in access to cART and HIV care historically. For example, FIOCRUZ-Brazil (which has had cART universally available since 1996) had the highest proportion of incident NADC diagnosed as well as high mortality following cancer diagnosis. Indeed, some studies from high-income settings with broad access to cART have also observed higher mortality for NADC than for ADC [22]. One important risk factor for incidence of and mortality after NADC is increasing age, an observation we also found in our study [32]. Older age alone has been associated with increased mortality in HIV and may be associated with poor response to HIV or cancer treatment or accumulation of other co-morbidities. Taken together, these findings underscore the epidemiologic changes observed in high-income settings of increased NADC incidence and mortality among an aging cohort of HIV patients that is also occurring in Latin America.

Lastly, our study is novel in its reporting of long term survival following cancer diagnosis in HIV patients in Latin America and showed important differences from what has been reported in high-income settings. Overall survival following cancer diagnosis in our study at one, three, and five years was 80%, 73%, and 68% respectively. In contrast, the five-year survival after cancer diagnosis was 54.5% following ADC diagnosis and approximately 65% following NADC diagnosis in the HIV Outpatient Study in the US [31]. Worm et al. reported five-year survival of 52.7% following NADC diagnosis in the D:A:D study [24]. These differences may be due to differences in specific types of cancers diagnosed (for instance, low rates of lung cancer observed in our cohort), patient characteristics (such as CD4 nadir or presence of co-infections, not included in our analysis), cancer treatment availability, or death ascertainment. Our study importantly adds to the understanding of cancer outcomes in HIV patients globally, including those from settings of limited resources and high prevalence of ADC.

There are limitations to our study to consider. First, though misclassification is a concern for any observational study, CCASAnet has gone to great lengths to maintain a high level of data quality including on-site audits of observational data collected [54]. It is possible that some cancer cases may be undiagnosed and therefore not included in this study due to differential rates of cancer screening or diagnosis. Second, our analysis was limited by high rates of missing laboratory data. This was addressed by multiple imputation in our analyses but is a common challenge for observational data in resource-limited settings. Third, other factors known to predict cancer outcomes were not included in this study, including cancer stage at diagnosis, cancer treatment, and smoking. Some studies from the US have suggested that HIV patients with cancer are less likely to receive appropriate cancer treatments compared to uninfected patients [55]. We did not have complete information available regarding cancer treatment received by patients and how this may differ by clinical site or patient characteristics such as cART use or immune status. Fourth, due to relatively low numbers of individual NADC diagnoses, we grouped the cancer diagnoses into ADC and NADC categories. Cancers are a heterogeneous mix of diseases and this approach, and the moderate numbers of events, may have limited our ability to detect clinical predictors of outcomes related to specific cancer diagnoses. Fifth, cancers diagnosed up to one year before the HIV diagnosis were included. It is probable that patients with ADC were more likely to be tested for HIV than patients with NADC, so there might be an under- representation of HIV patients with NADC in our cohort. Lastly, some studies have shown worse outcomes of NADC in HIV-positive people compared to the general population [1]. Our analysis lacks an HIV-uninfected population with which to compare survival outcomes following cancer diagnosis to evaluate the question of whether HIV patients in our region have comparably different outcomes than uninfected cancer patients.

## Conclusions

As one of the first studies to describe survival after cancer diagnosis in HIV-positive individuals from Latin America, this study provides valuable information to be used at the local level and shows the need of continuing investigation of cancer epidemiology to establish effective prevention and screening policies in this region. Future work will be strengthened by increasing observation time as patients with HIV and cancer live longer, incorporating additional information about specific cancer therapies in the region, and utilizing regional national cancer registries that may improve ascertainment and allow for comparisons with the general populations.
